# Preparation of Tough, Binder‐Free, and Self‐Supporting LiFePO_4_ Cathode by Using Mono‐Dispersed Ultra‐Long Single‐Walled Carbon Nanotubes for High‐Rate Performance Li‐Ion Battery

**DOI:** 10.1002/advs.202207355

**Published:** 2023-03-11

**Authors:** Mingyi Guo, Zengqiang Cao, Yukang Liu, Yuxiang Ni, Xianchun Chen, Mauricio Terrones, Yanqing Wang

**Affiliations:** ^1^ College of Polymer Science and Engineering Sichuan University Chengdu 610065 P. R. China; ^2^ School of Physical Science and Technology Southwest Jiaotong University Chengdu 610031 P. R. China; ^3^ Department of Physics Department of Chemistry Department of Materials Science and Engineering and Center for 2‐Dimensional and Layered Materials The Pennsylvania State University University Park PA 16802 USA

**Keywords:** binder‐free, lithium‐ion batteries, lithium iron phosphate, mono‐dispersed single‐walled carbon nanotubes, self‐supporting

## Abstract

Low‐contents/absence of non‐electrochemical activity binders, conductive additives, and current collectors are a concern for improving lithium‐ion batteries' fast charging/discharging performance and developing free‐standing electrodes in the aspects of flexible/wearable electronic devices. Herein, a simple yet powerful fabricating method for the massive production of mono‐dispersed ultra‐long single‐walled carbon nanotubes (SWCNTs) in *N*‐methyl‐2‐pyrrolidone solution, benefiting from the electrostatic dipole interaction and steric hindrance of dispersant molecules, is reported. These SWCNTs form a highly efficient conductive network to firmly fix LiFePO_4_ (LFP) particles in the electrode at low contents of 0.5 wt% as conductive additives. The binder‐free LFP/SWCNT cathode delivers a superior rate capacity of 161.5 mAh g^−1^ at 0.5 C and 130.2 mAh g^−1^ at 5 C, with a high‐rate capacity retention of 87.4% after 200 cycles at 2 C. The self‐supporting LFP/SWCNT cathode shows excellent mechanical properties, which can withstand at least 7.2 MPa stress and 5% strain, allowing the fabrication of high mass loading electrodes with thicknesses up to 39.1 mg cm^−2^. Such self‐supporting electrodes display conductivities up to 1197 S m^−1^ and low charge‐transfer resistance of 40.53 Ω, allowing fast charge delivery and enabling near‐theoretical specific capacities.

## Graphical abstract

A simple yet powerful fabricating method for the massive production of mono‐dispersed ultra‐long single‐walled carbon nanotubes (SWCNTs) in *N*‐methyl‐2‐pyrrolidone solution, benefiting from the electrostatic dipole interaction and steric hindrance of dispersant molecules, is reported. These SWCNTs form a highly efficient conductive network to firmly fix LiFePO_4_ particles in the electrode at low contents of 0.5 wt% as conductive additives.

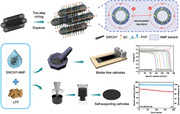

## 1 Introduction

To meet trends, such as the rise of flexible and wearable devices, significant advances in the energy storage capability of batteries are urgently required.^[^ ^1^ ^]^ In the dominant field of lithium‐ion batteries (LIBs), tremendous progress has been achieved in exploring the development of high‐capacity/rate capability electrode materials.^[^ ^2^ ^]^ Conventional processing of electrodes is realized by casting the slurries composed of active materials blended with additives, binders, and solvents on the metallic current collectors.^[^ ^3^ ^]^ The mechanical instabilities are caused by limited maximized mass loading, making the electrode impossible to prepare very thick even with polymeric binders.^[^ ^4^ ^]^ Besides, owing to the poor conductivity of electrode materials, a well‐dispersed continuous conductive network is highly desired for fast Li ions diffusion and charge transfer.^[^ ^5^ ^]^

Carbon materials, such as carbon nanotubes (CNTs) and graphene, with superior electrical conductivity and robust mechanical properties, have been proven to be a superior conductive additive,^[^ ^6^ ^]^ capable of achieving high capacity/rate capability electrode performance. However, the agglomeration of CNTs and graphene aroused due to their large specific surface area and *π*–*π* stack has made it difficult to construct a homogeneous conductive network in electrodes, especially when added at the low contents.^[^ ^7^ ^]^ ​In contrast to CNTs, large quantities of industrial graphene with few layers are currently often derived from mechanical or chemical stripping and have high oxygen‐containing functional groups and defects, which severely affect their electrical conductivity.^[^ ^8^ ^]^ CNTs, especially single‐walled carbon nanotubes (SWCNTs), tend to have more complete graphene sheets with excellent electrical conductivity.^[^ ^9^ ^]^ ​In addition, SWCNTs are more flexible than MWCNTs and the longer aspect ratio provides conditions for the construction of reliable physical entanglement. ​With the same amount of addition, more continuously interconnected networks of SWCNTs can anchor to the active material.

Besides exploring various kinds of electrode architectonics and materials to address these issues, many studies intensively focus on the preparation of binder‐free electrodes, which are proven to improve the rate capability of the electrode.^[^ ^10^ ^]^ Even though various binder‐free electrodes by the incorporation of CNTs to envelope the active electrode materials can achieve high capacity/rate capability,^[^ ^11^ ^]^ their preparations are still completed by depositing active particles on CNTs through the complicated spraying, evaporation, sol–gel, or electrochemical procedures.^[^ ^10^, ^12^ ^]^ Moreover, their insufficient mass loading of active materials and mechanical cracking of the as‐obtained electrodes are then arisen due to the self‐accumulation of CNTs, which cannot offer continuous enveloped networks and stable conductive skeleton during repeated charge/discharge.^[^ ^13^ ^]^ Finally, the inevitable use of metallic current collectors, occupying 10% weight of the entire battery system, not only decreases the gravimetric capacity and charge transfer efficiency of the batteries but also raises unexpected security concerns during mechanical deformation. Therefore, it is critical to explore fancy binder‐free, self‐supporting, and tough electrode materials with high capacity yet with remaining economic benefits.

In this work, it demonstrates a simple yet powerful fabricating method for the massive production of mono‐dispersed ultra‐long SWCNTs in *N*‐methyl‐2‐pyrrolidone (NMP) solution and the construction of the continuously interconnected SWCNT networks in the positive lithium iron phosphate (LFP) electrode. First, it was confirmed by molecular dynamics (MD) simulation that dispersant molecules with specific structures can be adsorbed on ultra‐long SWCNTs through intermolecular forces, and prevent ultra‐long SWCNTs from re‐agglomeration by electrostatic and steric hindrance. Compared with the SWCNTs powder, mono‐dispersed ultra‐long SWCNTs were easier to form a highly efficient conductive network in the electrode even with less addition amount from 5 to 0.5 wt% as conductive additives. In the absence of a binder, the LFP particles could be effectively fixed under the entanglement and wrapping of mono‐dispersed ultra‐long SWCNTs to form a stable structure, which greatly improved the specific capacity and stability of electrodes at high rates. At the contents of 3 wt% as conductive additives, the specific capacity of the electrode could reach 90.7 mAh g^−1^ at 20 C, with a 71.3% capacity retention after 500 cycles at 5 C. Furthermore, we demonstrate a hierarchical composite where the nanotubes arrange themselves into networked membranes that envelop the active LFP particles in the as‐prepared self‐supporting SWCNTs electrodes. The resulting self‐supporting electrode exhibits excellent bending resistance and compression in the electrolyte, which can bear at least 7.2 MPa tensile stress. These studies will undoubtedly provide an effective and practical strategy for the construction of efficient conductive networks favorable for electron/ion transport in tough, binder‐free, and self‐supporting electrodes.

## 2 Results and Discussion

### 2.1 Preparation and Molecular Dynamics Simulation of SWCNT Mono‐Dispersion

**Figure 1:**
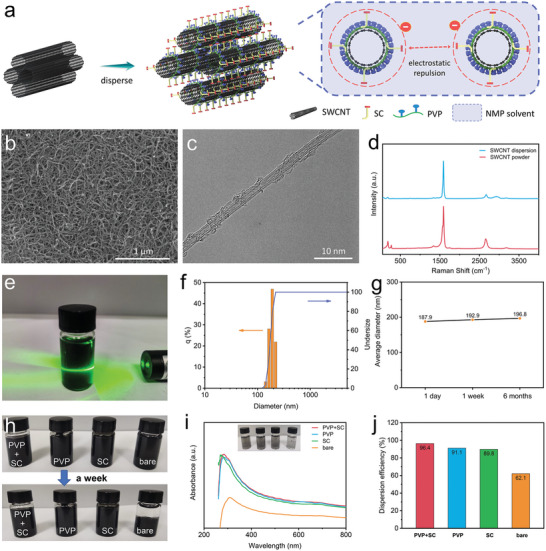
a) Schematic diagram of SWCNTs dispersion. b) SEM image of monodispersed SWCNTs in NMP solution. c) TEM image of monodispersed SWCNTs adsorbed dispersant. d) Raman spectra of SWCNTs dispersion and powder. e) Tyndall effect of SWCNT dispersion. f) Size distribution analysis of SWCNT dispersion. g) Average particle size. h) Photograph, i) UV–vis spectra, and j) dispersion efficiency of SWCNT‐NMP mixtures without dispersant and with different dispersants.

Battery performance is affected not only by the intrinsic properties of the active materials but also by the electrode structure.^[^ ^14^ ^]^ Well‐dispersed conductive networks in electrodes have been proven important for fast electron transport and charge transfer.^[^ ^15^ ^]^ In this work, ultra‐long SWCNTs were synergistically dispersed by dipole/dipole electrostatic interactions and steric hindrance, and the mono‐dispersion in NMP solution was prepared by a two‐step milling method, named as SWCNT‐NMP (**Figure** **1**a). The resulting dispersion in NMP was suction‐filtered into a film and dried, and the dispersion state of SWCNTs was observed by field emission scanning electron microscopy (SEM). The SWCNTs are staggered with each other, even under the action of force during the vacuum filtration process, they can still maintain an individual dispersion state with no obviously observed aggregates or tube bundles (Figure 1b). The transmission electron microscope (TEM) reveals an individual distribution of SWCNTs in the solvent, and the dispersant molecules are partially helically adsorbed on the surface of SWCNTs (Figure 1c). There is a very low‐intensity D peak and high‐intensity G peak in the bare SWCNT powders, indicating a very small amount of amorphous carbon or defects.^[^ ^16^ ^]^ After dispersion, the D peak disappears and the intensity of the G peak remains almost unchanged, which may be ascribed to the reason that mono‐dispersed SWCNTs are more inclined to a single distribution state and with an improved degree of order (Figure 1d).^[^ ^5^ ^]^ Meanwhile, as‐prepared mono‐dispersed SWCNTs in NMP solution appear as colloidal dispersion with obvious Tyndall phenomenon (Figure 1e). The particle size distribution was confirmed by nanoparticle size and zeta potential analyzer, as shown in Figure 1f, in which the particle size is normally distributed in a narrow range (D90 < 220 nm). The excellent stability of the dispersion was tested after the aqueous solution was left to stand for 1 week and 6 months, respectively, showing a negligible increase in the average particle size (Figure 1g). Figure 1h shows that the addition of dispersants prevents SWCNTs from re‐aggregating, while bare SWCNTs obviously subsided and agglomerated after being placed for a week. The UV–vis absorption spectrum was used to estimate the dispersion state of SWCNTs (Figure 1i). When the dispersion is diluted to 0.005% (mass fraction), the transmittance (at 500 nm of visible light) does not exceed 20%, showing homogenous dispersion. The dispersion efficiency was calculated after centrifugation at 10 000 rpm, and it was found that the polyvinylpyrrolidone (PVP) + sodium cholate (SC) mixed dispersant had the highest dispersion efficiency, reaching 96.4% (Figure 1j). It is attributed to the synergistic effect of the two dispersants. PVP increases the compatibility of SWCNTs in NMP and the dissociation of ${\mathrm{SO}}_{3}^{-}\mathrm{Na}$ ^+^ electron pairs in SC provide electrostatic repulsion, preventing SWCNTs from approaching each other, thus achieving stable mono‐dispersion.

**Figure 2:**
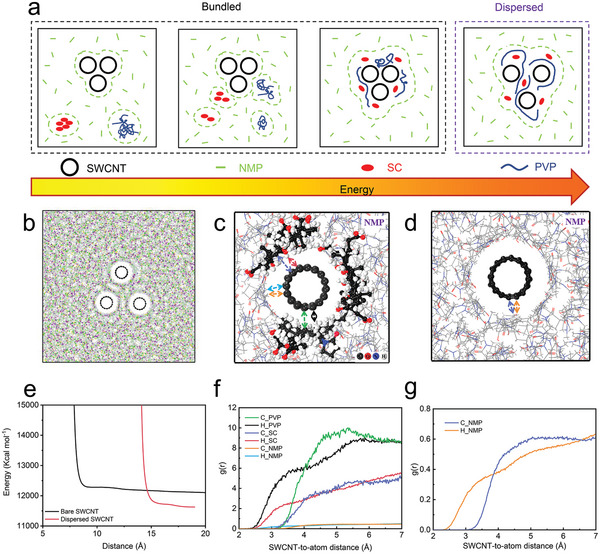
a) Schematic diagram of the process of disassembling bundled CNTs into individually dispersed nanotubes using dispersants. Snapshot of b) the atomistic model in MD simulation and the distances between atoms in NMP solution and the surface of c) dispersed SWCNT and d) bared SWCNT. e) Interaction energy changes during SWCNT approaching each other. RDF of C, H atoms to f) dispersed SWCNT and g) bared SWCNT.

The mono‐dispersion fabrication process for ultra‐long SWCNTs is shown in **Figure** **2**a. First, the dispersant molecules gradually approach the SWCNTs in the solvents and adsorb on their surfaces in the pre‐dispersion stage. With the input of external energy (such as grinding), the distance between the SWCNTs becomes larger, and the dispersant molecules gradually cover the entire surface of the individual SWCNTs, which promotes their further disassembling from the bundles and finally forms a uniform mono‐dispersed SWCNT dispersion.^[^ ^17^ ^]^ MD simulations were used to investigate the effect of dispersant molecules on the dispersion behavior of SWCNTs in the NMP solvent (Figure 2b). Under vacuum conditions, the interaction energy between the individual tubes is −97.1 kcal mol^−1^, showing the mutual attraction between nanotubes (Table S2, Supporting Information). To maintain the lowest energy in the system, the SWCNTs tend to bundle together. In the NMP‐filled conditions, the addition of dispersants decreases the interaction energy of SWCNTs and NMP, indicating that dispersant molecules can expel part of the NMP molecules around SWCNTs (Table S4, Supporting Information). The radial distribution function (RDF) also confirms that the C and H atoms of the dispersants are closer to the surface of SWCNTs (Figure 2f,g).^[^ ^18^ ^]^ We measured the energy changes of the system when two individual SWCNTs were brought into close proximity in NMP solvent (Figure S9, Supporting Information). The energy increases sharply when the distance between individual SWCNTs is reduced to 14.5 Å, indicating that the spacing cannot be further reduced, while the distance between bare SWCNTs can reach 7.5 Å (Figure 2e). MD simulations have shown that SWCNTs can be efficiently disassembled into individual SWCNTs in NMP under dipole/dipole electrostatic interactions of both SC and PVP, which are used as the driving force to disassemble the bundles of SWCNTs.^[^ ^19^ ^]^

### 2.2 Conductive Network Construction and Low‐Addition, High‐Rate Performance of LFP Cathode

**Figure 3:**
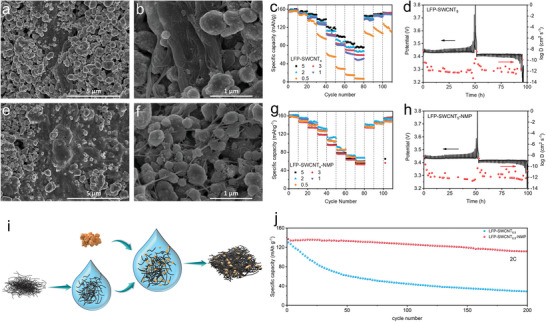
a,b) SEM image of LFP‐SWCNT_5_. c) Rate capability of LFP‐SWCNT*_x_*. d) GITT curves from LFP‐SWCNT_5_. e,f) SEM image of LFP‐SWCNT_5_‐NMP. g) Rate capability of LFP‐SWCNT*_x_*‐NMP. h) GITT curves from LFP‐SWCNT_5_‐NMP. i) Schematic diagram of the microcosm during the preparation of the cathodes. j) Comparison of cycling performance of cathode prepared from powders and dispersions, at 2 C, when *x* = 0.5.

In order to explore the effectiveness of constructing conductive networks of mono‐dispersed SWCNTs and their effects on the electrochemical performance of electrodes, LFP cathodes composed of bare SWCNTs powders and dispersions with various ratios of SWCNTs as conductive additives were prepared, named LFP‐SWCNT*_x_* and LFP‐SWCNT*_x_*‐NMP, respectively (*x* = 0.5, 1, 2, 3, 5, *x* represents the mass ratio of SWCNTs). As shown in **Figure** **3**a,b, large agglomerates and bundles are generated when using bare SWCNTs powders as conductive additives. It can be observed that there is a smooth and bare surface of LFP particles without any SWCNTs wrapping, indicating the inhomogeneous distribution of SWCNTs in the electrode. In contrast, for the LFP‐SWCNT‐NMP cathode, no obvious agglomerates or bundles can be observed (Figure 3e,f). The as‐prepared SWCNT dispersion produced by our method constructs an unbundled network in the whole regions of the electrode, even in the electrode regions with a higher density of SWCNTs. To evaluate the performance of conductive networks and gain the high‐performance electrode at low contents as conductive additives, a systematic study of LFP electrodes with various contents from 0.5 to 5 wt% of SWCNTs was performed. In LFP‐SWCNT*_x_*, the rate performance deteriorates significantly with decreasing conductive additive contents, especially at higher rates when electrons and ions need to be transported more efficiently (Figure 3c). However, through the construction of the mono‐dispersed SWCNTs conductive network, the resultant deterioration is not obvious as LFP‐SWCNT*_x_*. The rate performance of LFP‐SWCNT*_x_*‐NMP increases first and then decreases with the decrease of the addition amount, with an optimal addition amount of 2 wt% (Figure 3g). Moreover, the capacity gap of electrodes with different addition amounts was smaller, that is to say, the conductive network constructed by well‐dispersed SWCNTs in LFP electrodes was less affected by the addition amount.^[^ ^20^ ^]^ At low contents, the rate performance of LFP‐SWCNT_0.5_‐NMP is 72.9 and 59.7 mAh g^−1^ at high charge/discharge rates of 15 and 20 C, respectively, significantly more superior to that of LFP‐SWCNT_0.5_ (16.9 and 8.1 mAh g^−1^ at 15 and 20 C). It can be ascribed to the complete and well‐established stable conductive network by mono‐dispersed ultra‐long SWCNTs at low additions. The LFP‐SWCNT_0.5_‐NMP electrode shows a higher specific capacity retention of 81.4% after 200 cycles at a high charge/discharge rate of 2 C, compared with 22.2% retention for the LFP‐SWCNT_0.5_ electrode (Figure 3j). The charge transfer resistance (*R*_ct_) of the LFP‐SWCNT_0.5_‐NMP electrode was calculated as 62.8 Ω, which was lower than that of the other composites (87.4, 86.7, 64.8, and 71.8 Ω for LFP‐SWCNT_5_‐NMP, LFP‐SWCNT_3_‐NMP, LFP‐SWCNT_2_‐NMP, and LFP‐SWCNT_1_‐NMP, respectively) and much lower than that of all the LFP‐SWCNT electrodes (Figures S10d and S11d, Supporting Information).

The galvanostatic intermittent titration technique (GITT) was further used to investigate the fast electron/ion transporting behaviors of the LFP electrode. The kinetic behavior of LFP‐SWCNT_5_ and LFP‐SWCNT_5_‐NMP were investigated by GITT and the Li^+^ diffusion coefficient (*D*_GITT_) was calculated based on the following equation^[^ ^21^ ^]^

(1) $$D=4L^{2}/\pi \tau {\left(\Delta E_{s}/\Delta E_{t}\right)}^{2}$$

where *L* is the Li^+^ diffusion length (cm), *τ* is the relaxation time (s), Δ*E*_s_ is the steady‐state voltage change (V) of the current pulse, and Δ*E*_t_ is the potential change (V) during the cross‐current pulse after removing the iR drop.

In the GITT process, the quiescent voltage of LFP‐SWCNT_5_ and LFP‐SWCNT_5_‐NMP was 3.43 V for all quiescent steps during charging and discharging, showing the occurrence of Li^+^ intercalation/deintercalation at this potential. The average diffusion coefficients of Li^+^ in LFP‐SWCNT_5_ and LFP‐SWCNT_5_‐NMP were calculated to be 1.29 × 10^−10^ and 1.61 × 10^−10^ cm^2^ s^−1^, respectively, indicating that the LFP‐SWCNT_5_‐NMP has better ion transport behavior and smaller electrode polarization.^[^ ^15c^ ^]^

### 2.3 Preparation and Performance of Binder‐Free Electrode (LFP‐SWCNT*_x_*‐BF)

**Figure 4:**
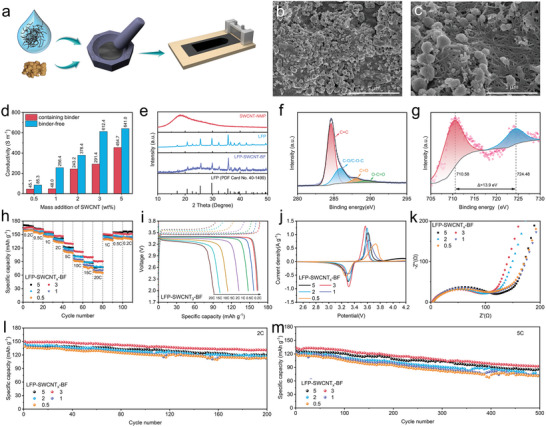
a) Schematic diagram of the process of preparing cathodes by casting method. b,c) SEM images of LFP‐SWCNT*_x_*‐BF. d) Conductivity of cathodes with and without PVDF binder. e) XRD patterns of SWCNT‐NMP, LFP, and LFP‐SWCNT‐BF. XPS high‐resolution f) C 1s and g) Fe 2p spectrum for LFP‐SWCNT_3_‐BF. h) Rate performance. i) Galvanostatic charge and discharge (GCD) curves of LFP‐SWCNT_3_‐BF. j) CV curves at a scan of 0.2 mV s^−1^. k) Nyquist plots. Cycling performance at l) 2 C and m) 5 C.

In the conventional preparation of electrodes, an electrochemically inert polyvinylidene difluoride (PVDF) binder needs to be added to connect the active material, conductive agent, and current collector.^[^ ^11^, ^22^ ^]^ In addition to decreasing the electron transfer rate, they also increase the weight of the electrode and thus reduce the overall energy density.^[^ ^10a^ ^]^ Herein, a binder‐free electrode was prepared, named LFP‐SWCNT_x_‐BF (**Figure** **4**a), where the LFP particles were wrapped and fixed by the interconnected ultra‐long SWCNT networks, providing physical bonding and stable support during the charging and discharging.^[^ ^4^ ^]^ As shown in Figure 4b,c, the distribution of ultra‐long SWCNTs in the cathode was relatively homogeneous with no observed SWCNT bundles. The individual ultra‐long SWCNTs were interconnected to form a 3D network, and thus, physical binding was achieved by wrapping and pinning LFP active particles. Moreover, the excellent mechanical strength of SWCNTs provides a certain strengthening for the cathode structure, which is beneficial for maintaining the stability of the electrode structure. As shown in Figure 4d, the cathode mixed slurry was coated on the insulated PET film for conductivity measurement. Under the same amount of addition, the electrode without PVDF binder had significantly higher conductivity than the electrode with PVDF binder. As confirmed by X‐ray diffraction (XRD), the introduction of dispersants during disassembling of the bundles into individual tubes had no adverse effect on the crystal structure of the LFP, and would not affect the redox reaction during the charging and discharging process (Figure 4e). To characterize the chemical composition and state of the LFP‐SWCNT*_x_*‐BF cathodes, X‐ray photoelectron spectroscopy (XPS) was used for testing. A wide spectrum shows the peaks of Li 1s, P 2p, O 1s, and Fe 2p elemental compositions, without typical peaks of F 1s (Figure S13, Supporting Information). The C 1s peaks at 284.6, 285.8, 288, and 289.5 eV can be ascribed to the C=C, C—O/C—O—C, C=O, and O—C=O bonds, respectively (Figure 4f).^[^ ^15^, ^22^ ^]^ In the high‐resolution XPS spectrum of Fe 2p, the diffraction peaks of Fe 2p_3/2_ and Fe 2p_1/2_ are located at 710 and 724.1 eV, respectively, with a peak spacing of 13.9 eV, consistent with the LFP feature (Figure 4g).^[^ ^15^, ^21^ ^]^

Due to the absence of PVDF binder, all the LFP‐SWCNT*_x_*‐BF cathodes have a higher specific capacity than the results mentioned above and LFP‐SWCNT_5_‐BF has a closer capacity to the theoretical value of LFP, reaching 169.2 mAh g^−1^ at 0.2 C. With the decrease of the SWCNTs contents from 5 to 0.5 wt%, there is no obvious deterioration in the rate performance. LFP‐SWCNT_3_‐BF (mass loading ≈1.5 mg cm^−2^) exhibited the best rate capacities of 161.5, 156.1, 146.8, 130.2, 111.9, 99.9, and 90.7 mAh g^−1^, at 0.5, 1, 2, 5, 10, 15, and 20 C, respectively (Figure 4h). Moreover, the voltage profiles for typical charge/discharge cycles at 0.2, 0.5, 1, 2, 5, 15, and 20 C are shown in Figure 4i. However, the binder‐free LFP‐SWCNT_3_‐BF electrodes do not contain PVDF, so only 3 wt% of the non‐conducting dispersant molecules remain. As a result, the direct contact area between the active material and the SWCNTs exposure is larger, and thus the active material shows the best performance at higher addition amount (3 wt%). The potential intervals between the two redox peaks of the LFP‐SWCNT*_x_*‐BF electrodes with contents of 0.5, 1, 2, 3, and 5 wt% were 0.464, 0.305, 0.280, 0.257, and 0.304 V, respectively, showing that the LFP‐SWCNT_3_‐BF electrode has the lowest internal potential, which can be ascribed to the increasing Li^+^ diffusion depth and thus reduced polarization (Figure 4j). ​At the addition of 3 wt%, a sufficiently continuous and efficient 3D conductive network with enough pores can be formed in the positive electrode, which provides faster Li^+^ diffusion behavior during electrochemical reactions, and thus the shortening of the diffusion time to the surface of the active material. The charge transfer resistance (*R*_ct_) of the LFP‐SWCNT_3_‐BF electrode was calculated as 83.88 Ω, which is lower than that of the other composites (Figure 4k). According to GITT, the average diffusion coefficient of LFP‐SWCNT_3_‐BF was calculated to be 8.41 × 10^−10^ cm^2^ s^−1^ (Figure S10, Supporting Information). After the removal of the binders, the coverage area of the electrical insulating substance inside the electrode is reduced, which can greatly improve the electron transport efficiency and the electrochemical reaction rate. The LFP‐SWCNT_3_‐BF electrode shows a higher specific capacity retention of 87.4% after 200 cycles at 2 C and 71.3% after 500 cycles at 5 C, respectively, compared with other cathodes (Figure 4l,m). These results demonstrated that it can achieve comparable or higher values than most references listed in Table S5, Supporting Information, with a lower content as a conductive additive and provide a practical strategy for the application of conductive additives in high‐rate charge/discharge LIBs.

### 2.4 Performance of Tough, Binder‐Free, and Self‐Supporting Electrode (LFP‐SWCNT‐SS)

**Figure 5:**
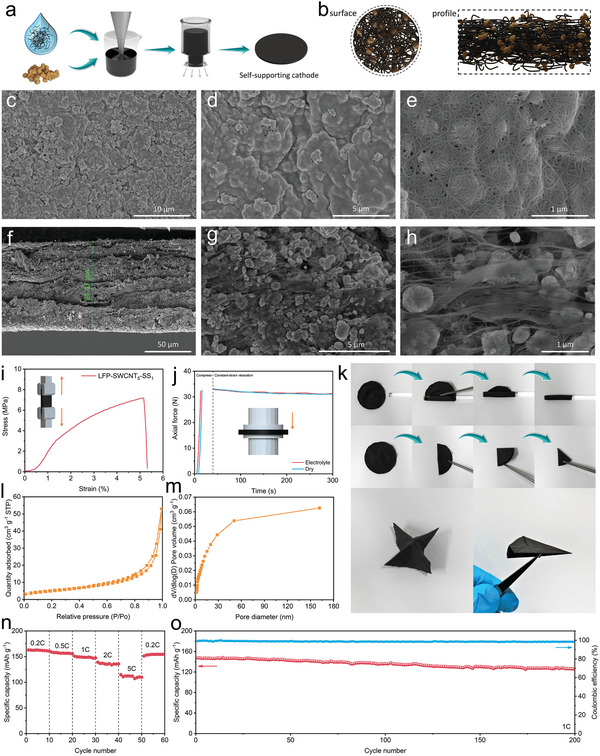
a) Schematic diagram of the self‐supporting cathodes by vacuum filtration. b) Structural diagram of the surface and profile of self‐supporting cathodes. SEM images of c–e) the surface and f–h) the cross profile of LFP‐SWCNT_5_‐SS. i) Stress–strain curve. j) Microenvironment stability test under compression and relaxation. k) Bending performance test. l) N_2_ adsorption/desorption isotherms and m) pore size distributions. n) Rate and o) cycling performance.

The slurry with lower viscosity is hard to cast onto the collector with a blade owing to the absence of binder, thus causing a low LFP mass loading in the cathodes (<3 mg cm^−2^).^[^ ^15^, ^20^ ^]^ In order to prepare the thick electrodes with a density over 3 mg cm^−2^, vacuum filtration was adopted to get the self‐supporting cathodes with higher LFP mass loadings (>8 mg cm^−2^), named LFP‐SWCNT_x_‐SS. The synthesis method is illustrated in **Figure** **5**a. Different from the traditional coating process, the organic solvent can be collected and reused after filtration, so it is more environmentally friendly and economical.^[^ ^13b^ ^]^ Reducing the amount of SWCNTs resulted in brittle membrane electrodes due to the absence of a metal collector. Therefore, 5 wt% was chosen as the addition amount of SWCNTs in the self‐supporting electrodes. Various high mass loadings of self‐standing, binder‐free LFP‐SWCNT_5_‐SS cathodes were obtained (mass loadings ≈8.1, 18.7, 26.5, and 39.1 mg cm^−2^, respectively). With the increase in electrode thickness, there was negligible shrinkage in the radial direction (Figure S17, Supporting Information). The surface (Figure 5c–e) and cross‐sectional (Figure 5f–h) microstructures of the LFP‐SWCNT_5_‐SS cathode were observed, and as prepared mono‐dispersed ultra‐long SWCNTs can form a long‐range film‐like network by tightly wrapping adjacent LFP active particles or by establishing connections between the active particles as conductive bridges.^[^ ^10^, ^23^ ^]^ Due to the force of vacuum filtration, the individual SWCNTs or film‐forming bundles were roughly distributed parallel to the surface (Figure 5f–h).

The elemental distribution of the self‐standing, binder‐free LFP‐SWCNT_5_‐SS electrode was measured by mapping C, O, Fe, and P elements, revealing a uniform distribution in the cross‐sectional electrode (Figure S16, Supporting Information). N_2_ adsorption/desorption isotherms are shown as a shape of V with distinct H_3_‐typed hysteresis loops, and a high specific surface area of 18.7 m^2^ g^−1^ and a pore volume of 0.045 cm^3^ g^−1^, providing rapid and uniform diffusion of ions in the porous LFP cathode and accelerating electrochemical reactions at the electrode/electrolyte interface (Figure 5l,m).^[^ ^3c^ ^]^ XRD patterns of LFP and LFP‐SWCNT_5_‐SS electrodes were matched with PDF Card No. 40–1499, showing the absence of an impurity phase (Figure S21, Supporting Information). The self‐supporting cathode exhibits strong bending resistance and durability, which can be curled and folded into any shape such as “airplane” without any damage (Figure 5k).

Tensile experiments also show that the self‐supporting electrode LFP‐SWCNT_5_‐SS has excellent mechanical properties, which can withstand at least 7.2 MPa stress and 5% strain (Figure 5i). The electro‐rheological properties of the LFP‐SWCNT_5_‐SS cathodes at both dry and wet states were investigated, which are important parameters to evaluate whether the electrodes can maintain good structures under pressure in the electrolyte.^[^ ^24^ ^]^ The compression and relaxation curves of LFP‐SWCNT_5_‐SS in the wet state are very similar to those in the dry state (Figure 5j). Electrochemical tests were performed in half‐cells without collectors. Rate performance was obtained for the LFP‐SWCNT_5_‐SS electrode, showing high capacitance of 162.9, 158.8, 150.6, 139.1, and 114.7 mAh g^−1^, at 0.2, 0.5, 1, 2, and 5 C, respectively, and much lower *R*_ct_ of 40.53 Ω compared with that of LFP‐SWCNT_5_‐BF (107.3 Ω) (Figure 5n). The half‐cell was charged and discharged at 1 C for 200 cycles to test the stability of the cathode, and the cycling performance at 1 C shows that the LFP‐SWCNT_5_‐SS electrode has a retention of 85.2% with a coulombic efficiency of ≈99% (Figure 5O).

## 3 Conclusion

In this paper, we report on a simple yet powerful method for the massive production of mono‐dispersed ultra‐long SWCNTs in NMP solution, benefiting from electrostatic dipole interactions and steric hindrance of the dispersant molecules. Based on SWCNT‐NMP, LFP cathodes with homogeneously dispersive efficient conductance networks were fabricated and compared with those prepared from agglomerated powder, showing that conductive networks constructed from mono‐dispersed SWCNTs with reduced additive amounts are more stable. ​Furthermore, the electrochemically inert PVDF was removed and the binder‐free LFP‐SWCNT‐BF was fabricated by directly mixing with LFP particles and SWCNT‐NMP and casting on the collector, showing promising electrochemical properties of a specific capacity of 90.7 mAh g^−1^ at 20 C and retention of 81.4% after 200 cycles at 2 C. ​ ​In addition, a flexible self‐supporting cathode LFP‐SWCNT_5_‐SS without collector and binder was fabricated by vacuum filtering the mixed slurry of LFP particles and SWCNT‐NMP. LFP‐SWCNT_5_‐SS exhibits strong bending resistance of curling and folding, tensile property of withstanding stress of at least 7.2 MPa and strain of 5%, a high capacitance of 139.1 mAh g^−1^ at 2 C, and much low impedance of *R*_ct_ of 40.53 Ω, benefiting from the high specific surface area of 18.7 m^2^ g^−1^ and conductivities of 1197 S m^−1^. ​These studies undoubtedly provide an effective and practical strategy for the construction of the most efficient conducting network for the application of high‐performance binder‐free and flexible self‐supporting electrodes.

## 4 Experimental Section

### Preparation of Dispersion

SWCNTs (Tuball, OCSiAl, diameter <2 nm) were prepared as mono‐dispersion via the following steps: i) 3.3 g of the as‐purchased SWCNT powders, 1.65 g of SC (Acros), and 1.65 g of PVP (TCI Chemicals) were pre‐dispersed into 1000 mL NMP using a continuously operating bead mill system for 24 h; ii) the pre‐dispersed slurry was further grinded using a bead mill (Multi Lab DYNO‐Mill, 0.6 mm zirconium beads) for 16 h to ensure the SWCNTs were dispersed individually. ​The resulting homogeneous monodispersed SWCNT slurry was named SWCNT‐NMP.

### Preparation of Cathodes

The components of the cathodes with a total solid content of 100 mg included 5 wt% PVDF as the binder, the different SWCNT powder mass ratios ranging from 5, 3, 2, 1, and 0.5 wt%, and the corresponding LiFePO_4_ mass ratios 90, 92, 93, 94, and 94.5wt %, named as LFP‐SWCNT*_x_*, in which *x* (mg) represented the mass of SWCNTs per 100 mg of the cathode. In a similar way, the cathode that SWCNT powder was replaced with dispersion named LFP‐SWCNT*_x_*‐NMP. Furthermore, in order to reduce the content of inactive substances, the addition of PVDF was removed, and the corresponding binder‐free cathode was named LFP‐SWCNT*_x_*‐BF. The details are shown in Table S1, Supporting Information. Meanwhile, a self‐supported and flexible LFP cathode with high mass loading without collector and binder was prepared with SWCNT dispersion, named LFP‐SWCNT_5_‐SS (*x* = 5). The cathode slurry of LFP‐SWCNT*_x_*, LFP‐SWCNT*_x_*‐NMP, and LFP‐SWCNT*_x_*‐BF was obtained by grinding the mixture of LFP powder and conductive additive for 30 min, and then adding PVDF solution of NMP (solid content was 20 mg mL^−1^), after that, fully mixed for 30 min. The cathodes were prepared by depositing them on a carbon‐coated aluminum foil and dried in a vacuum at 80 °C for 12 h. Self‐supported cathode slurry was obtained by stirring magnetic stirring for 30 min, the mixture of LFP powder and SWCNT dispersion, which was diluted to 50 mL, and then stirred for 30 min after ultrasonic processing (on/off = 2s/2s at 5% power) in the Ultrasonic Homogenizer (JY92‐IIDN, 900 W) for 10 min. The self‐supported cathodes were fabricated by vacuum filtration and dried to completely remove the solvent. For the specific mass additions of each component of preparation, refer to Table S1, Supporting Information.

### Materials Characterizations

The morphology and the distribution of the components in the dispersion and the electrode were obtained with field emission SEM (FEI Nova Nano 450) and energy dispersive spectrometer (EDS). Individual SWCNTs in the dispersion were observed by TEM observations (FEI Talos F200S Super‐X) at an accelerating voltage of 200 kV. Degrees of dispersion was confirmed via size distribution analysis using a nanoparticle size and zeta potential analyzer (BeNano 90 Zeta, Bettersize Instruments Ltd.). Elemental composition was examined by XPS (Thermo Scientific K‐Alpha) with an Al K*α* X‐ray source. XRD was acquired on a Smart Lab. The specific surface area and pore structures of electrodes were analyzed using Nitrogen adsorption/desorption by the Brunauer–Emmett–Teller (BET, Micromeritics ASAP 2460) method at 77 K.

### Electrochemical Measurements

All the cathodes used for electrochemical measurements were punched as 12 mm diameter discs. The as‐prepared cathodes were assembled into Coin‐type (CR2032) half‐cells, which used lithium foil as the counter electrode, a glass fiber membrane (Whatman, GF/D) as the separator, and 1 m LiPF_6_ in 1 m LiPF_6_ in a mixture of ethyl carbonate/dimethyl carbonate (EC/DMC, 1:1, in volume). The electrochemical performances of obtained batteries were tested on the Neware battery tester in the voltage range of 2–3.8 V (vs Li/Li^+^) for LFP electrodes at different current densities (the current density of 1 C was ≈0.17 A g^−1^) on the Neware battery tester at room temperature (25 ± 2 °C). All the batteries were activated for five cycles at 0.2 C before the rate and cycling performance were tested. Cyclic voltammetry (CV) measurements were performed on an electrochemical workstation (CHI660E) at a scan rate of 0.1 mV s^−1^. The electrochemical impedance spectrum (EIS) tests were also conducted over a frequency range from 100 kHz to 0.01 Hz.

### Molecular Dynamics Simulation

MD simulations were performed using the LAMMPS package. The potential function polymer consistent force field (PCFF) was used to describe the various bonding and non‐bonding interactions involving dispersants and SWCNTs.^[^ ^17^ ^]^ The simulation process was carried out in a box of size 6 × 6 × 5 nm. The box space was filled with NMP molecules, and the SWCNTs were placed in the center. Two systems were considered, one contained dispersants and the other one was without dispersants. For the specific parameters of the simulation, refer to Tables S2–S4, Supporting Information.

## Conflict of Interest

The authors declare no conflict of interest.

## Supporting information

Supporting Information

## Data Availability

The data that support the findings of this study are available in the supplementary material of this article.
