# Supplementary material for: Preparation of Tough, Binder‐Free, and Self‐Supporting LiFePO4 Cathode by Using Mono‐Dispersed Ultra‐Long Single‐Walled Carbon Nanotubes for High‐Rate Performance Li‐Ion Battery
Source: Adv Sci (Weinh). 2023 Mar 11;10(13):2207355. doi: 10.1002/advs.202207355 (PMC10161069; doi:10.1002/advs.202207355)
Supplement: Supplementary file 1 — Supporting Information [file ADVS-10-2207355-s001.pdf]

ADVANCED SCIENCE
Open Access

Supporting Information

for *Adv. Sci.*, DOI 10.1002/advs.202207355

Preparation of Tough, Binder-Free, and Self-Supporting $LiFePO_4$ Cathode by Using Mono-Dispersed Ultra-Long Single-Walled Carbon Nanotubes for High-Rate Performance Li-Ion Battery

*Mingyi Guo, Zengqiang Cao, Yukang Liu, Yuxiang Ni, Xianchun Chen, Mauricio Terrones and Yanqing Wang**

Supporting Information

Preparation of Tough, Binder-Free and Self-Supporting $LiFePO_4$ Cathode by Using Mono-Dispersed Ultra-Long Single-Walled Carbon Nanotubes for High-Rate Performance Li-Ion Battery

Mingyi Guo[a], *Zengqiang Cao*[b], *Yukang Liu*[a], *Yuxiang Ni*[b], *Xianchun Chen*[a], *Mauricio Terrones*[c] *and Yanqing Wang*[a,]*

[a] *College of Polymer Science and Engineering, Sichuan University, Chengdu, 610065, China*

[b] *School of Physical Science and Technology, Southwest Jiaotong University, Chengdu 610031, China*

[c] *Department of Physics, Department of Chemistry, Department of Materials Science and Engineering and Center for 2-Dimensional and Layered Materials, The Pennsylvania State University, University Park, PA 16802, USA*

Table S1. LFP cathodes with different conductive additives

Name		Mass in slurry (mg)			
Series	x	LFP	SWCNT	PVDF	Dispersant
$LFP-SWCNT_x$	5	90	5	5	-
	3	92	3	5	-
	2	93	2	5	-
	1	94	1	5	-
	0.5	94.5	0.5	5	-
$LFP-SWCNT_x-NMP$	5	90	5	5	5
	3	92	3	5	3
	2	93	2	5	2
	1	94	1	5	1
	0.5	94.5	0.5	5	0.5
$LFP-SWCNT_x-BF$	5	90	5	-	5
	3	94	3	-	3
	2	96	2	-	2
	1	98	1	-	1
	0.5	99	0.5	-	0.5
$LFP-SWCNT_5-SS$	-	90	5	-	5
	-	180	10	-	10
	-	270	15	-	15
	-	360	20	-	20

Annotation: x represents the mass ratio of SWCNT in the cathode.

Table S2. Interaction energy between individual components in vacuum

	SWCNT-SWCNT	PVP-PVP	SC-SC	NMP-NMP
Interaction energy (kcal mol^{-1})	-97.10	-59.47	-39.69	-12.96

Table S3. The number of molecules contained in each system

	PVP (count)	SC (count)	SWCNT (count)	NMP (count)	Density (g cm^{-3})
Bared SWCNT	0	0	3	968	1.037
Dispersed SWCNT	3	15	3	833	1.038

Table S4. Interaction energy between SWCNTs and other components

	SWCNT-PVP	SWCNT-SC	SWCNT-NMP
Bared SWCNT	-	-	-357.51
Dispersed SWCNT	-32.54	-55.20	-270.05

Table S5. Performance of LFP electrode

Type of conductive additives	Mass ratio	Rate performance	Ref
Super P, MWCNT, GO	10, 0.5, 0.5 wt%	101 mAh g^{-1} at 5 C	[1]
Acetylene black, MWCNT	8, 4 wt%	72 mAh g^{-1} at 10 C	[2]
SWCNT	5 wt%	105 mAh g^{-1} at 10 C	[3]
N-MWCNT	5 wt%	48 mAh g^{-1} at 10 C	[4]
MWCNT	2.78 wt%	115 mAh g^{-1} at 10 C	[5]
S-MWCNT	4wt%	143 mAh g^{-1} at 2 C	[6]
Super P, MWCNT	12, 1.5 wt%	72 mAh g^{-1} at 10 C	[7]
MWCNT, rGO	4, 4 wt%	88 mAh g^{-1} at 10 C	[8]

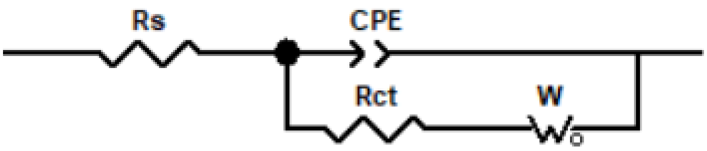

Figure S1. Model of electrochemical impedance spectroscopy.

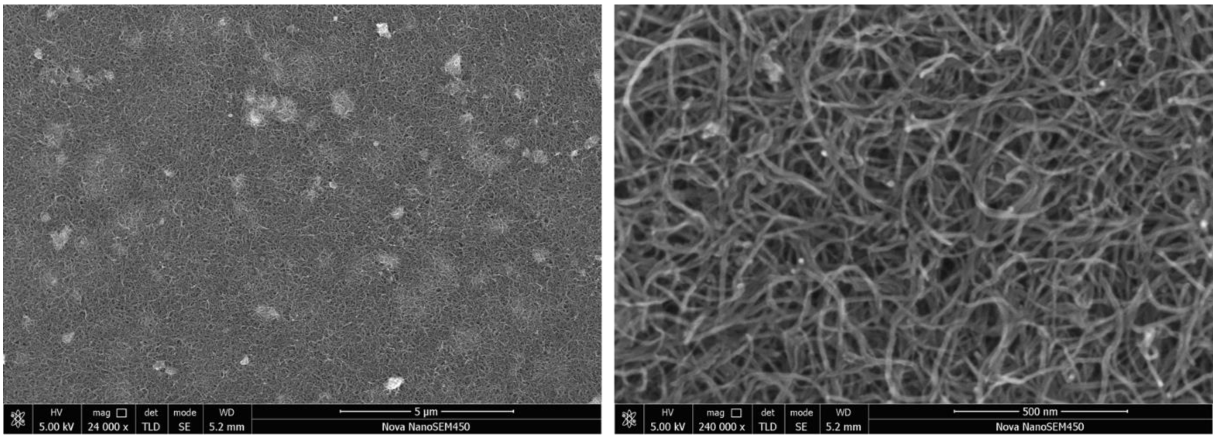

Figure S2. SEM of SWCNT-NMP derived membrane after vacuum filtration and drying.

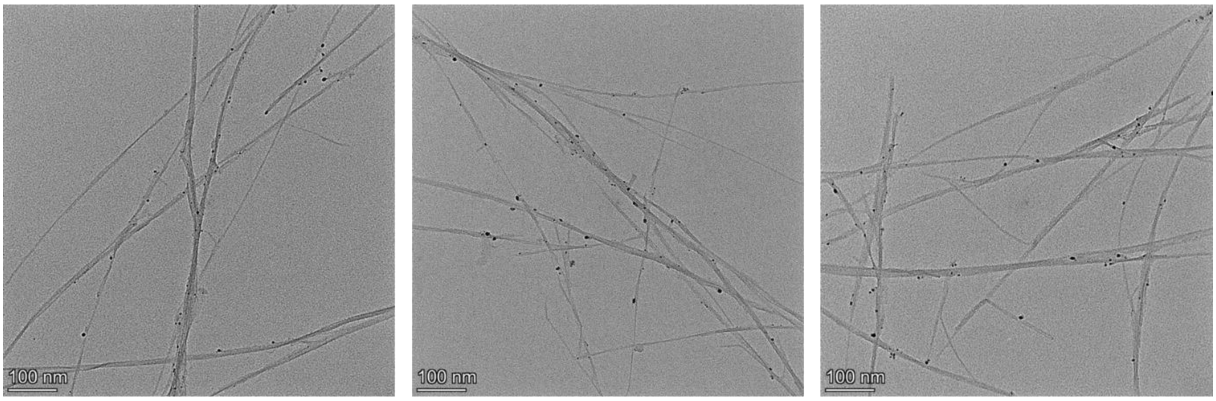

Figure S3. TEM of SWCNT-NMP.

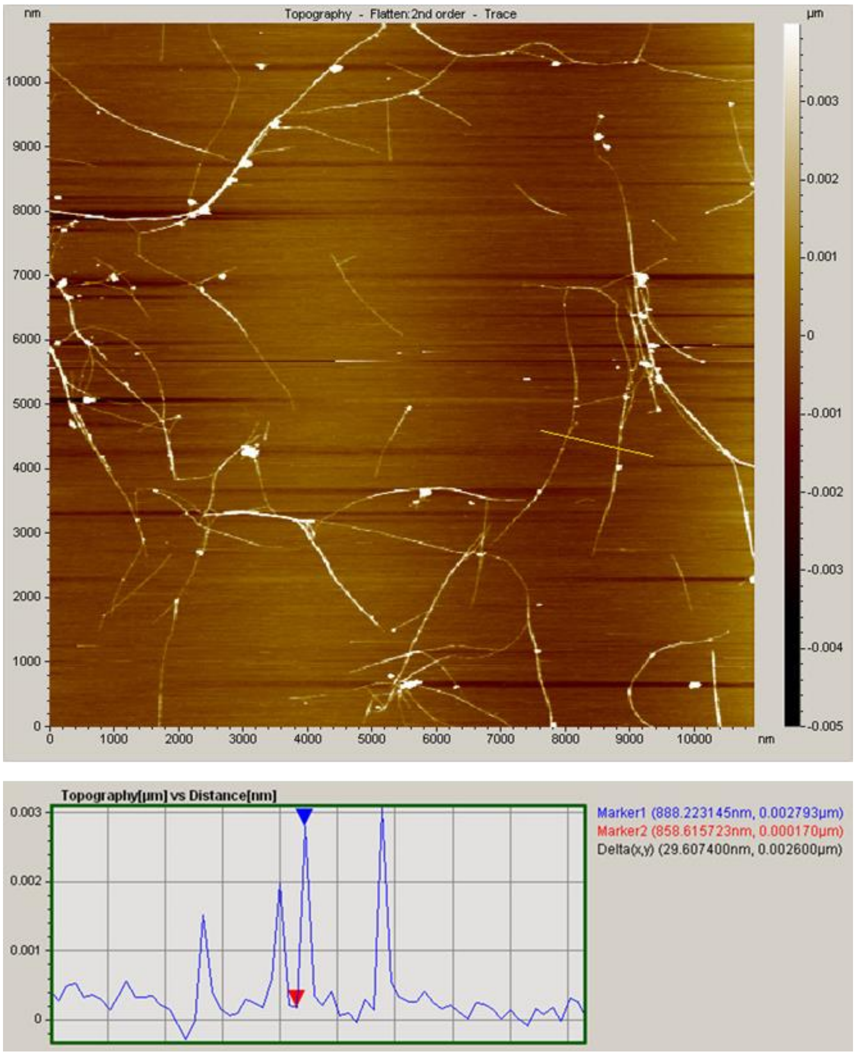

Figure S4. AFM of SWCNT-NMP.

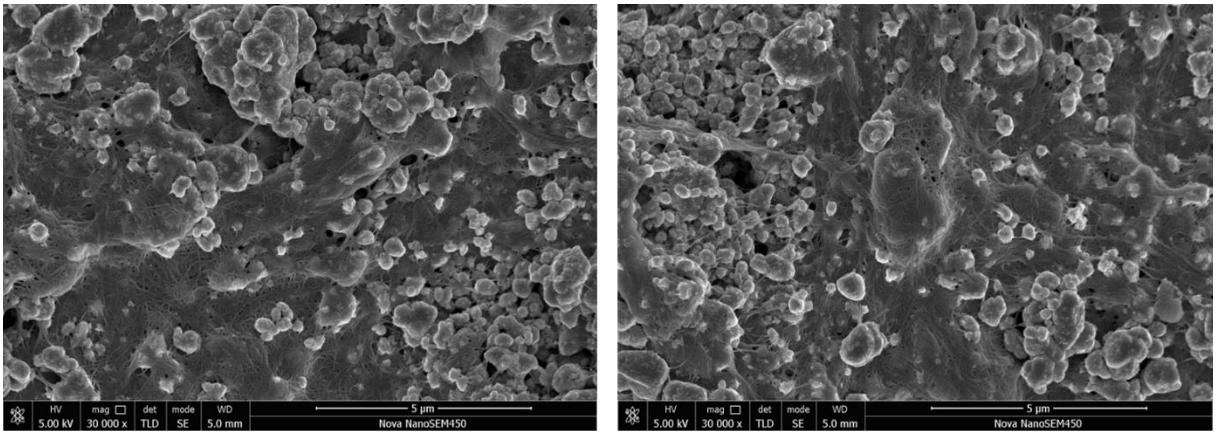

Figure S5. SEM of surface of LFP-SWCNT-NMP cathode.

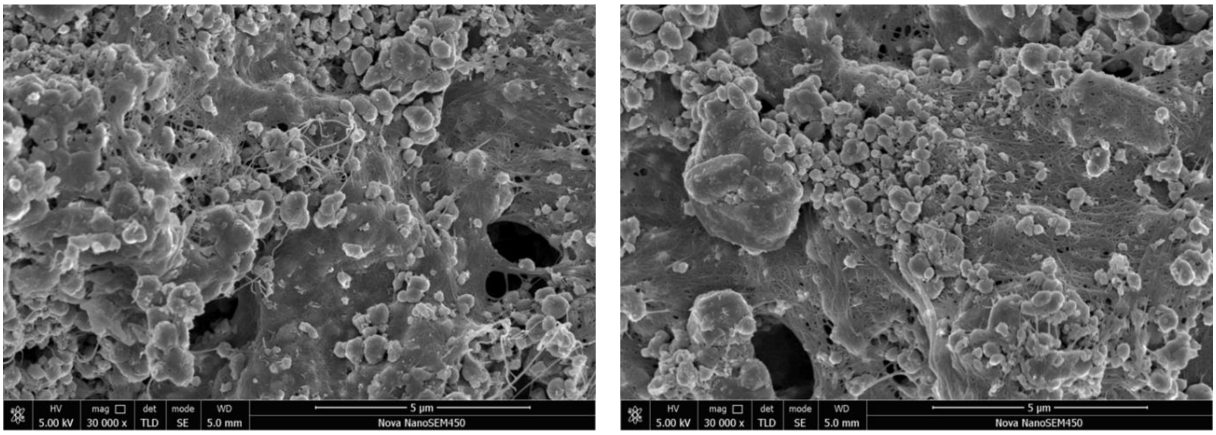

Figure S6. SEM of surface of LFP-SWCNT-BF cathode.

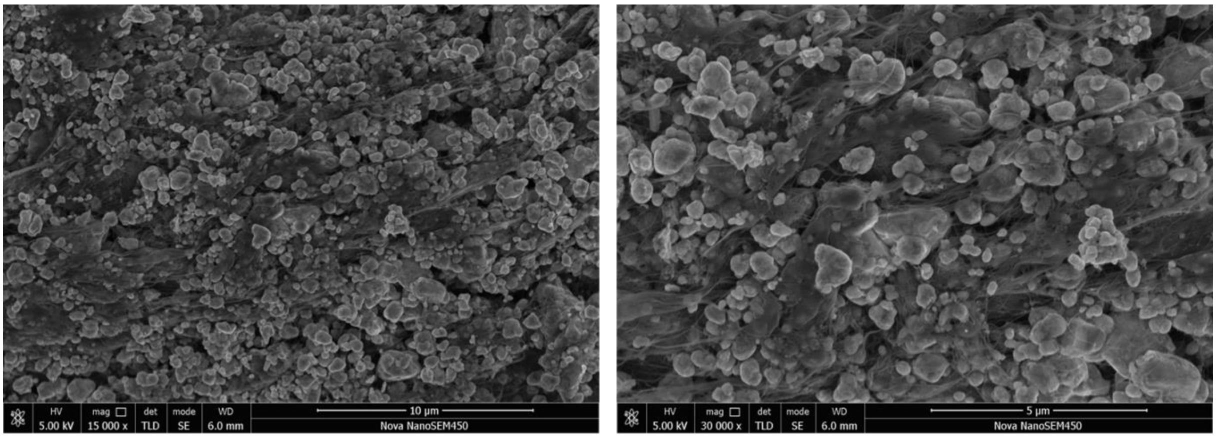

Figure S7. SEM of surface of LFP-SWCNT-BF cathode.

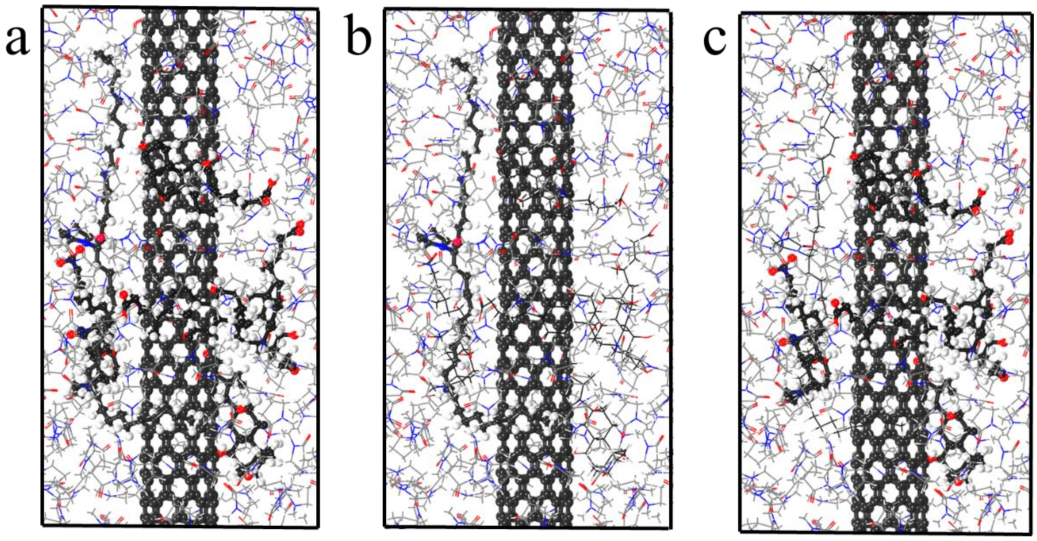

Figure S8. Snapshot of molecular dynamics processes.

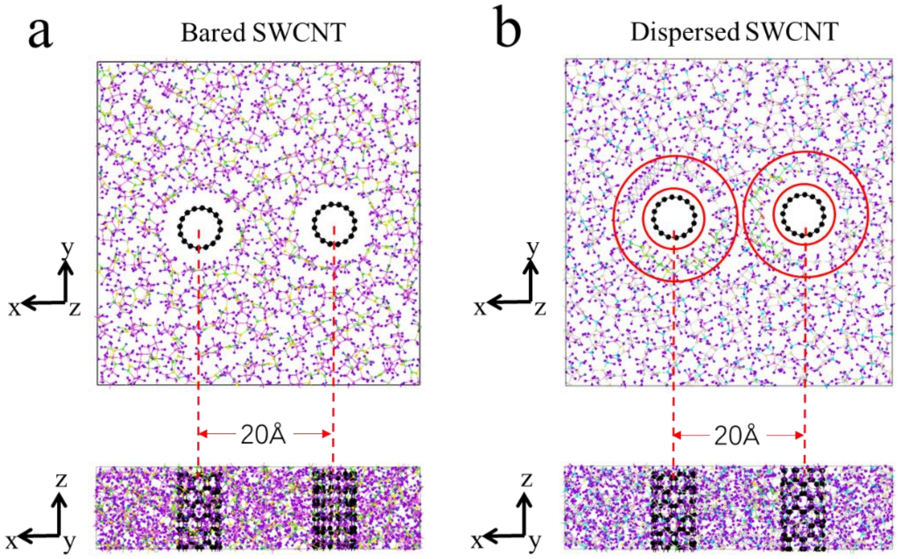

Figure S9. Schematic diagram of molecular dynamics simulation of SWCNT spacing variation of a) Bared SWCNT and b) Dispersed SWCNT.

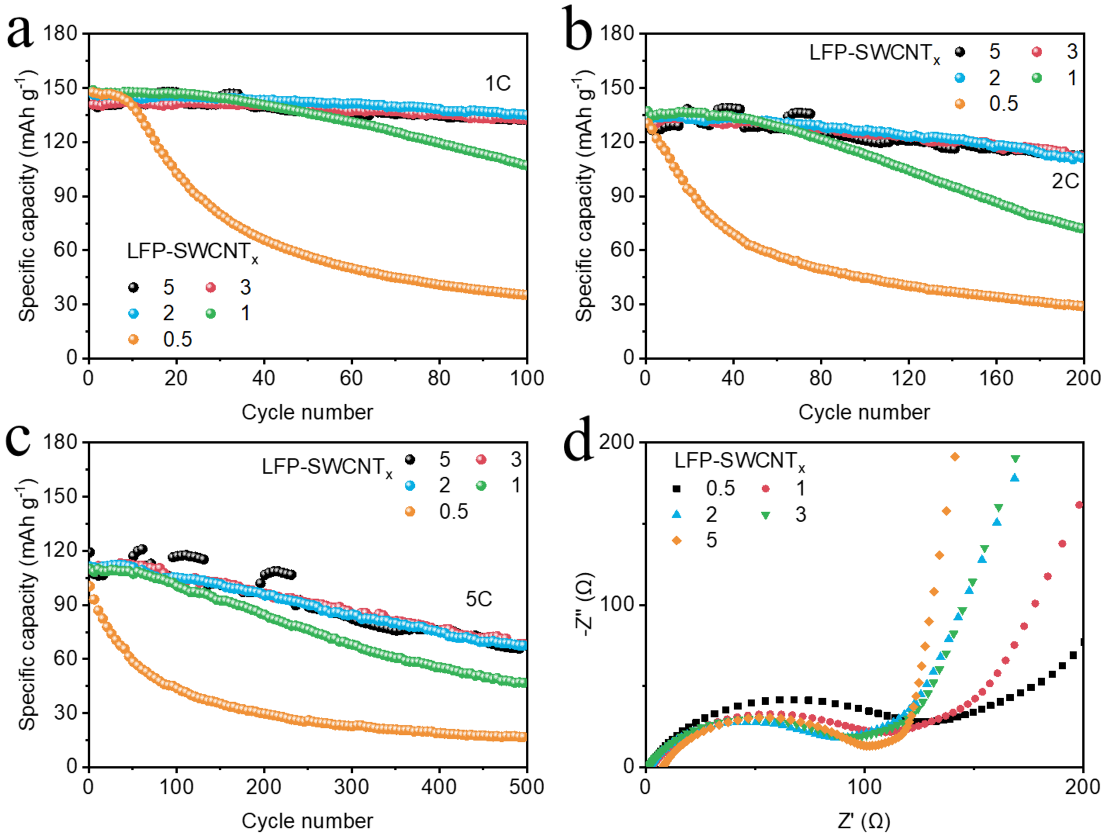

Figure S10. Cycle performance of LFP-SWCNT$_x$, at the current density of a) 1 C, b) 2 C and

c) 5 C respectively. d) Nyquist curve of LFP-SWCNT$_x$.

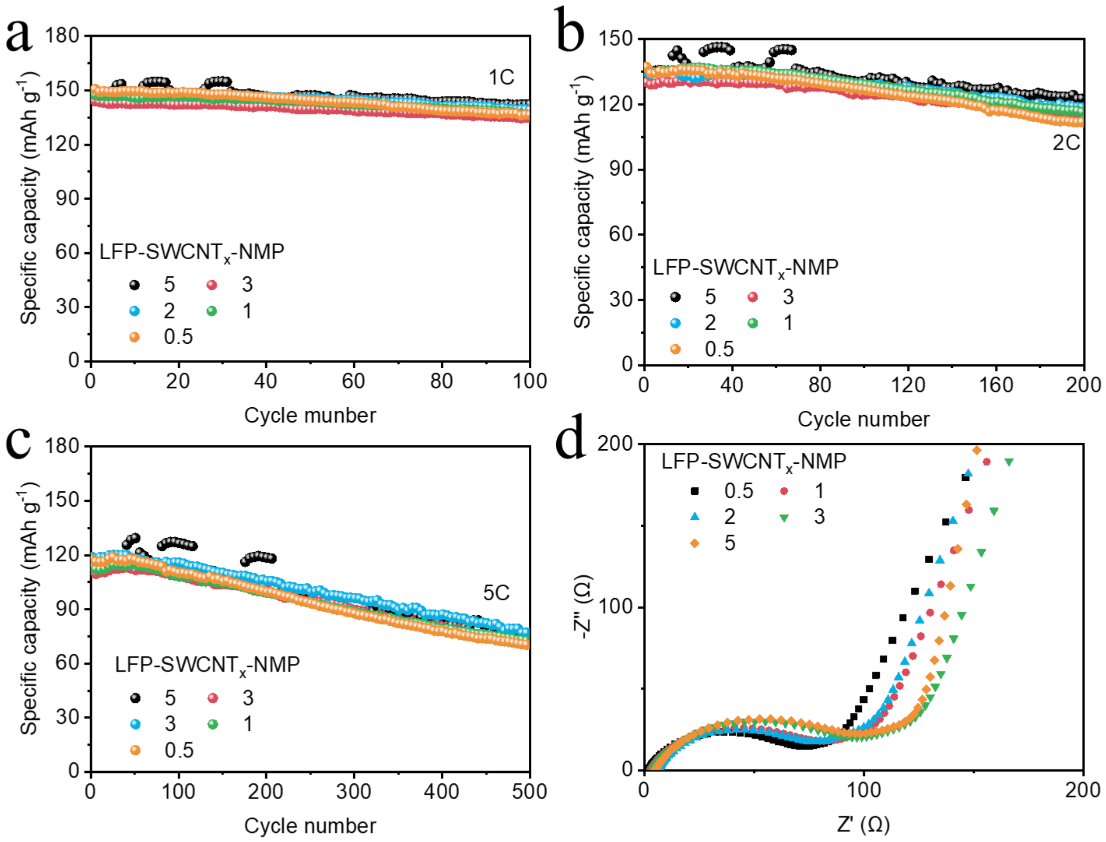

Figure S11. Cycle performance of LFP-SWCNT$_x$-NMP, at the current density of a) 1 C, b) 2 C and c) 5 C respectively. (d) Nyquist plot of LFP-SWCNT$_x$-NMP.

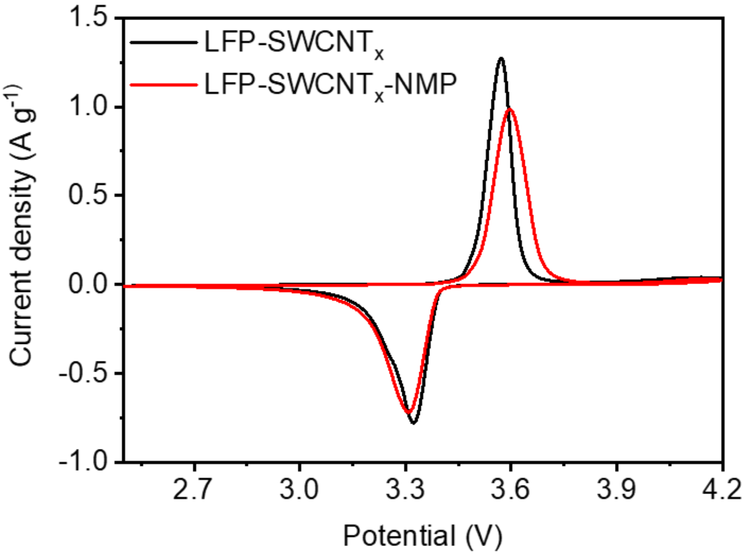

Figure S12. CV curves comparison of LFP-SWCNT$_5$ and LFP-SWCNT$_5$-NMP.

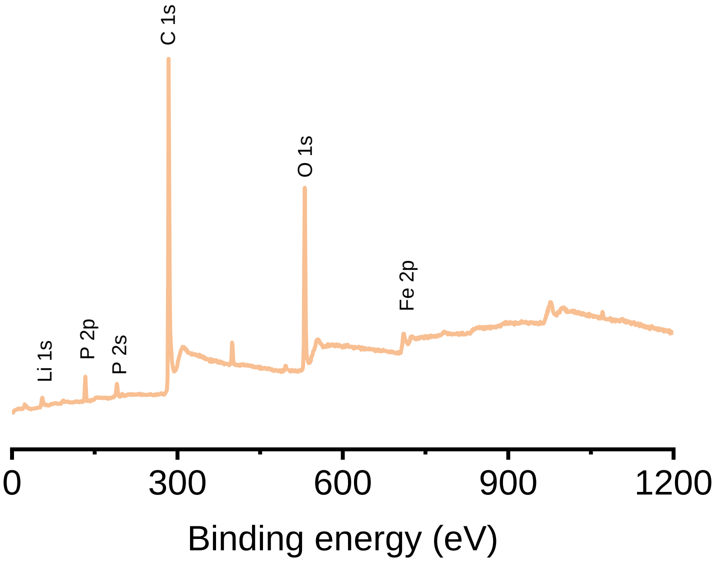

Figure S13. XPS wide spectrum of LFP-SWCNT$_3$-BF.

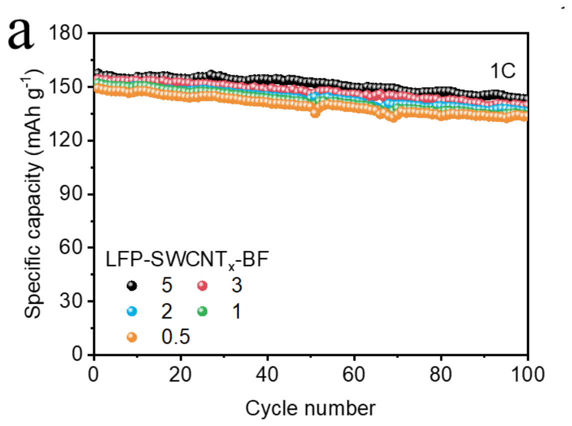

Figure S14. Cycle performance of LFP-SWCNT$_x$-BF at 1 C.

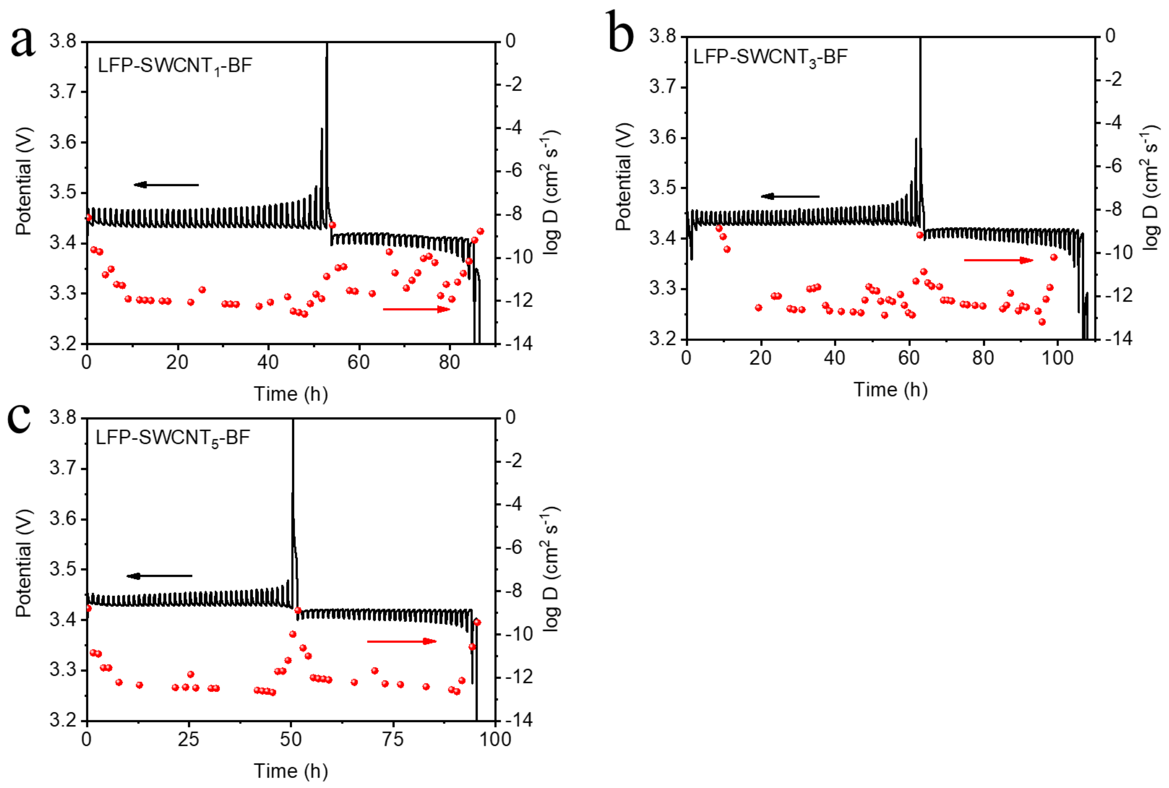

Figure S15. Galvanostatic intermittent titration technique curves of LFP-SWCNT$_x$-BF.

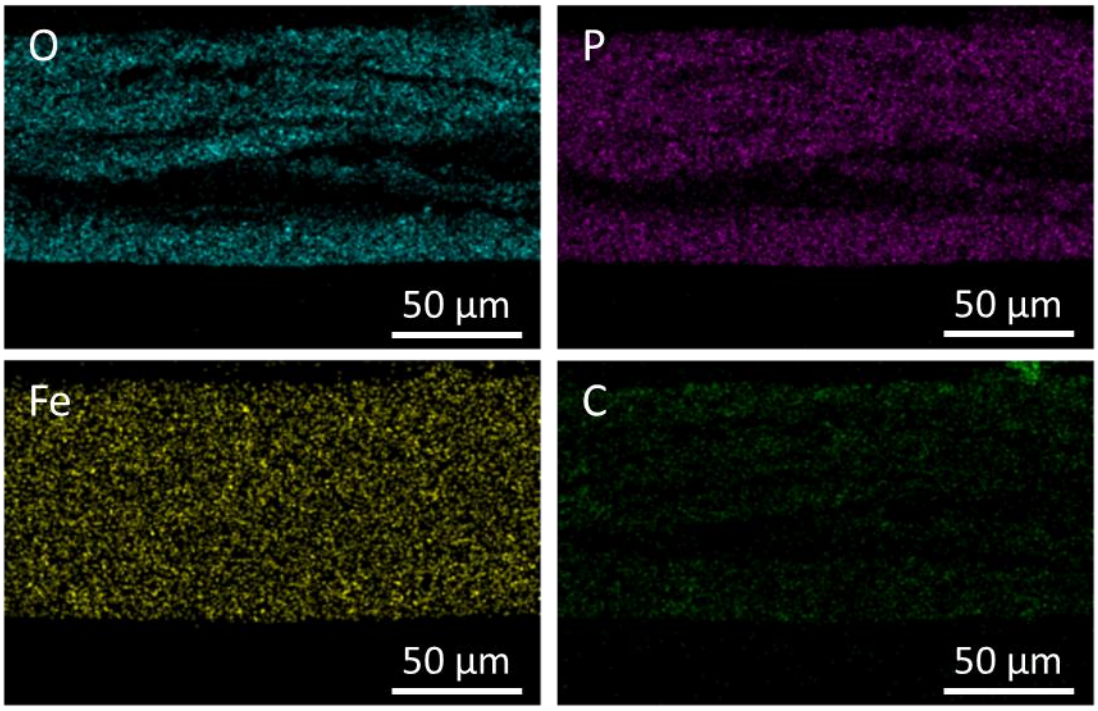

Figure S16. EDS mapping with O, P, Fe and C elemental distribution on the cross profile.

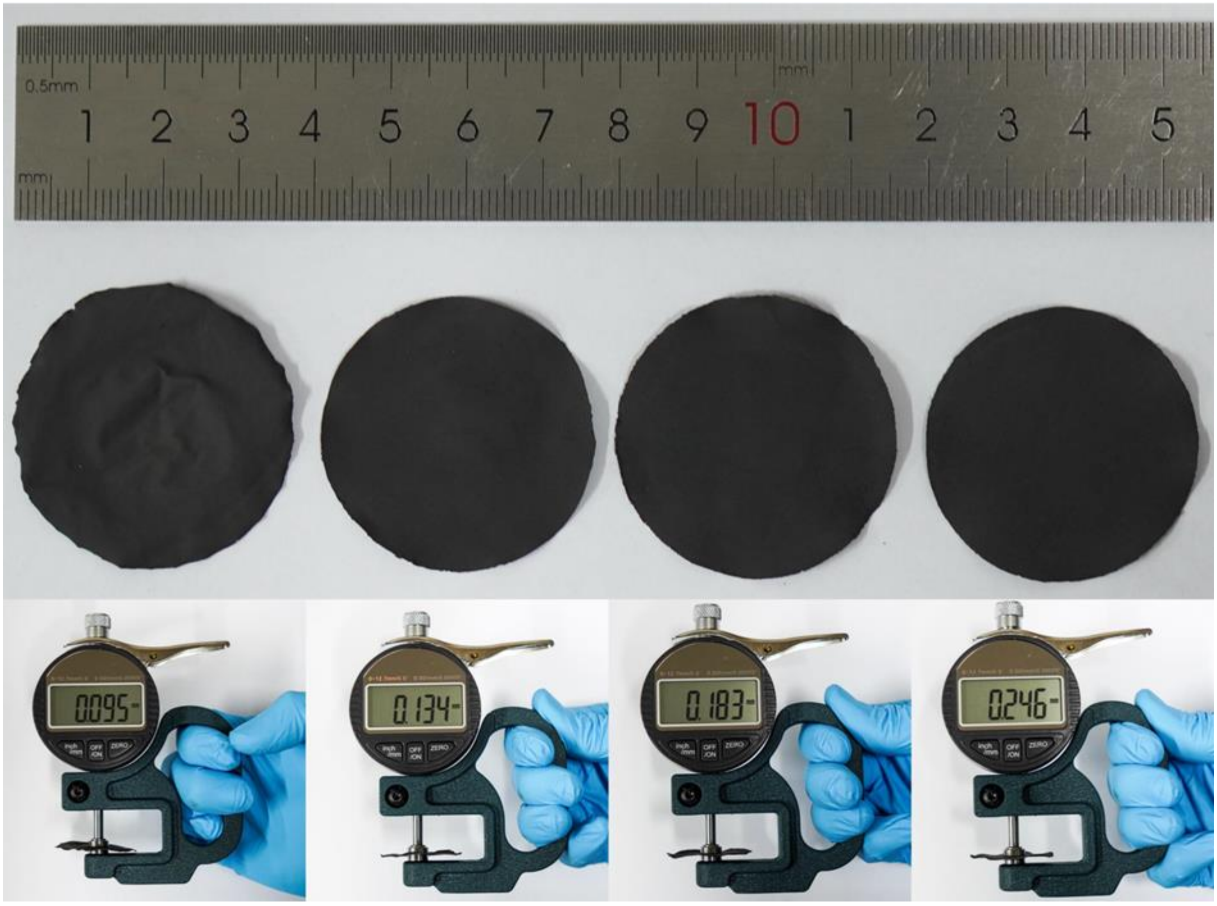

Figure S17. The size and thickness of cathodes with different LFP mass loading.

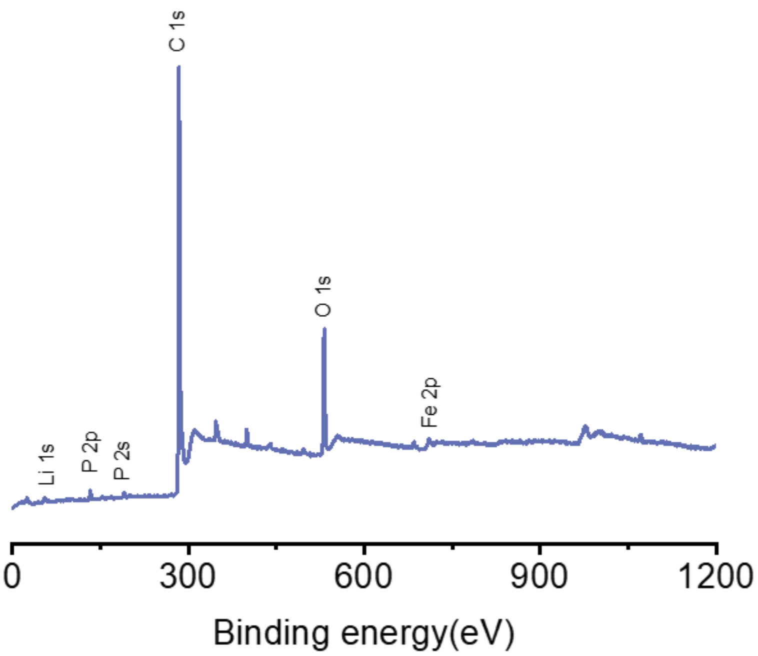

Figure S18. XPS wide spectrum of LFP-SWCNT$_5$-SS.

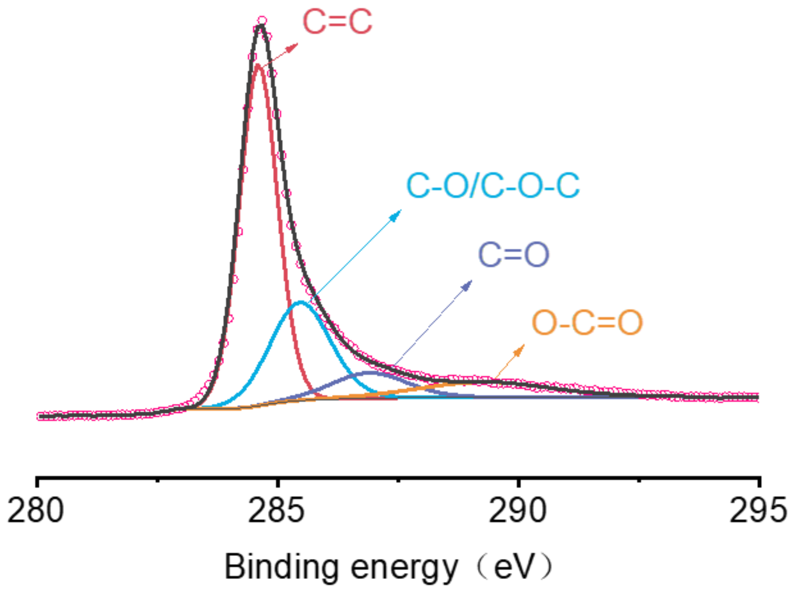

Figure S19. XPS high-resolution C 1s spectrum of LFP-SWCNT$_5$-SS.

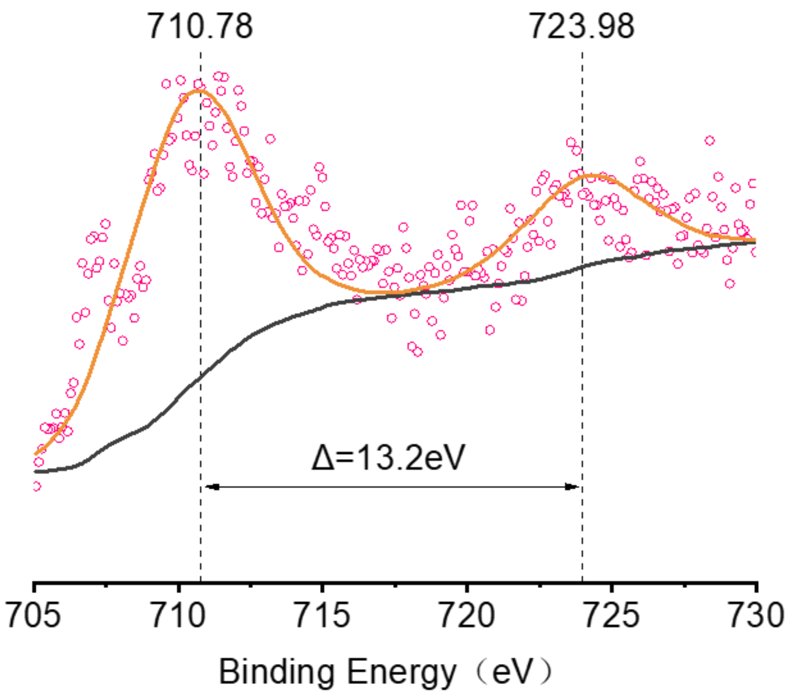

Figure S20. XPS Fe 2p spectrum of LFP-SWCNT$_5$-SS.

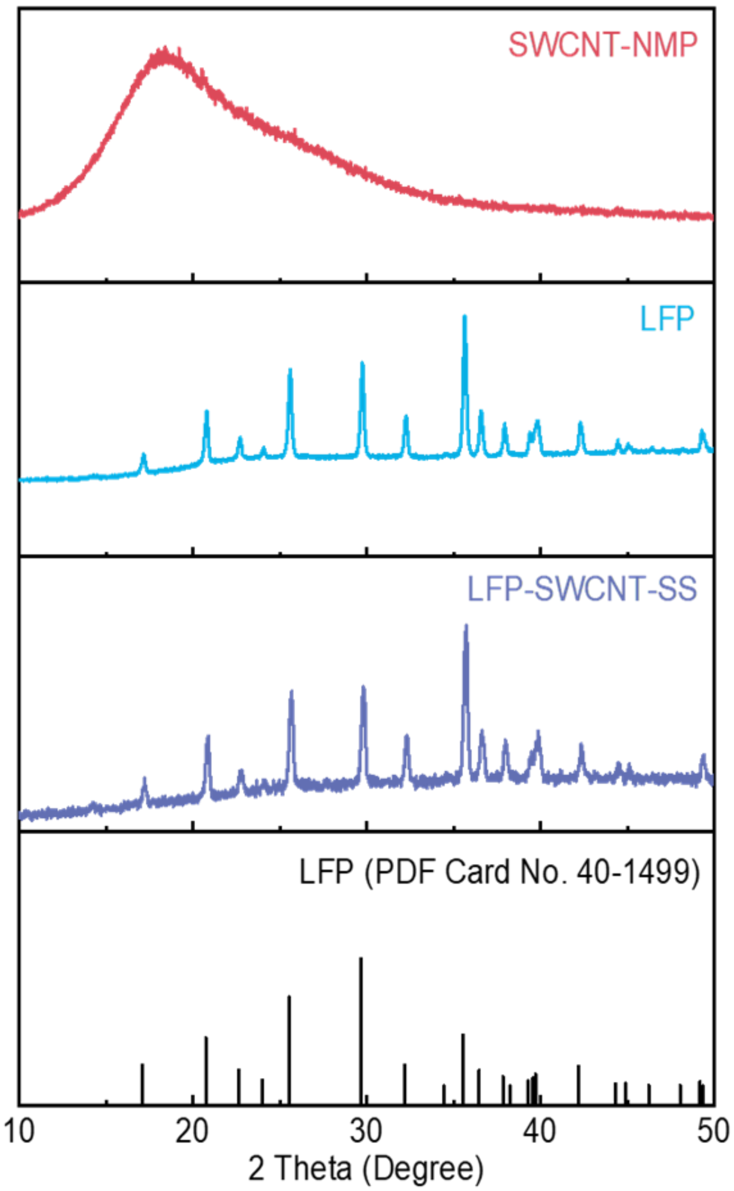

Figure S21. XRD patterns of SWCNT-NMP, LFP, LFP-SWCNT$_5$-SS.

Reference

[1] Y. Cai, D. Huang, Z. Ma, H. Wang, Y. Huang, X. Wu, Q. Li, *Electrochim. Acta* **2019**, *305*, 563, https://doi.org/10.1016/j.electacta.2019.02.114.

[2] L. Tan, Q. Tang, X. Chen, A. Hu, W. Deng, Y. Yang, L. Xu, *Electrochim. Acta* **2014**, *137*, 344, https://doi.org/10.1016/j.electacta.2014.06.015.

[3] S. Rousselot, P. Antitomaso, L. Savignac, S. Généreux, L. W. Taylor, T. Bibienne, M. Pasquali, S. B. Schougaard, M. Dollé, *Electrochim. Acta* **2020**, *349*, https://doi.org/10.1016/j.electacta.2020.136418.

[4] J. Yang, J. Wang, X. Li, D. Wang, J. Liu, G. Liang, M. Gauthier, Y. Li, D. Geng, R. Li, X. Sun, *J. Mater.*

Chem. **2012**, *22* (15), https://doi.org/10.1039/c2jm30380a.
[5] B. Wang, T. Liu, A. Liu, G. Liu, L. Wang, T. Gao, D. Wang, X. S. Zhao, *Adv. Energy Mater.* **2016**, *6* (16), https://doi.org/10.1002/aenm.201600426.
[6] C. Qi, X. Ma, G. Ning, X. Song, B. Chen, X. Lan, Y. Li, X. Zhang, J. Gao, *Carbon* **2015**, *92*, 245, https://doi.org/10.1016/j.carbon.2015.04.028.
[7] X. Tu, Y. Zhou, Y. Song, *Appl. Surf. Sci.* **2017**, *400*, 329, https://doi.org/10.1016/j.apsusc.2016.12.220.
[8] J. Lu, Y. Zhou, T. Jiang, X. Tian, X. Tu, P. Wang, *Ceram. Int.* **2016**, *42* (1), 1281, https://doi.org/10.1016/j.ceramint.2015.09.063.
